# CXCR4 Antagonist AMD3100 Modulates Claudin Expression and Intestinal Barrier Function in Experimental Colitis

**DOI:** 10.1371/journal.pone.0027282

**Published:** 2011-11-03

**Authors:** Xian-Ming Xia, Fang-Yu Wang, Ju Zhou, Kai-Feng Hu, Su-Wen Li, Bing-Bing Zou

**Affiliations:** 1 Department of Gastroenterology and Hepatology, The Fourth Affiliated Hospital of Anhui Medical University, Hefei, Anhui Province, People's Republic of China; 2 Department of Gastroenterology and Hepatology, Jinling Hospital, Nanjing, Jiangsu Province, People's Republic of China; 3 Department of General Surgery, The Fourth Affiliated Hospital of Anhui Medical University, Hefei, Anhui Province, People's Republic of China; Charité-University Medicine Berlin, Germany

## Abstract

Ulcerative colitis is a gastrointestinal disorder characterized by local inflammation and impaired epithelial barrier. Previous studies demonstrated that CXC chemokine receptor 4 (CXCR4) antagonists could reduce colonic inflammation and mucosal damage in dextran sulfate sodium (DSS)-induced colitis. Whether CXCR4 antagonist has action on intestinal barrier and the possible mechanism, is largely undefined. In the present study, the experimental colitis was induced by administration of 5% DSS for 7 days, and CXCR4 antagonist AMD3100 was administered intraperitoneally once daily during the study period. For *in vitro* study, HT-29/B6 colonic cells were treated with cytokines or AMD3100 for 24 h until assay. DSS-induced colitis was characterized by morphologic changes in mice. In AMD3100-treated mice, epithelial destruction, inflammatory infiltration, and submucosal edema were markedly reduced, and the disease activity index was also significantly decreased. Increased intestinal permeability in DSS-induced colitis was also significantly reduced by AMD3100. The expressions of colonic claudin-1, claudin-3, claudin-5, claudin-7 and claudin-8 were markedly decreased after DSS administration, whereas colonic claudin-2 expression was significantly decreased. Treatment with AMD3100 prevented all these changes. However, AMD3100 had no influence on claudin-3, claudin-5, claudin-7 and claudin-8 expression in HT-29/B6 cells. Cytokines as TNF-α, IL-6, and IFN-γ increased apoptosis and monolayer permeability, inhibited the wound-healing and the claudin-3, claudin-7 and claudin-8 expression in HT-29/B6 cells. We suggest that AMD3100 acted on colonic claudin expression and intestinal barrier function, at least partly, in a cytokine-dependent pathway.

## Introduction

Ulcerative colitis (UC) is a gastrointestinal disorder characterized by inflammatory response and mucosal damage [1]. Uncontrolled local inflammation disrupts the epithelial lining, resulting in mucosal edema and ulceration, and even crypt abscess in the bowel wall [2]. In healthy individuals, the intestinal barrier is constituted of an intact layer of epithelial cells, act as the gateway restricting uncontrolled entry of luminal antigens [3]. Intestinal epithelial barrier is maintained by intracellular junctional complexes, such as tight junctions (TJ), adherent junctions, and desmosomes [4]. TJ form an intra-membranous fence between the apical and lateral plasma membrane domains, and intimately involved in both paracellular permeability and cell polarity [3], [5].

TJ is composed of transmembrane proteins, such as claudins and occludin, and cytosolic proteins, such as ZO-1. Claudins, which is the major integral membrane proteins forming the continuous TJ strands, interact in a tissue-specific manner to form a charge-selective and size-selective barrier, and predominantly contribute to epithelial barrier function [6]–[10]. In UC, epithelial barrier function is impaired. Previous investigations by freeze fracture electron microscopy demonstrated a reduction of TJ strands in UC, which is considered to be a possible cause of barrier dysfunction [4], [11]. Additionally, the disrupted morphology of TJ is often the result of changes in TJ protein expression [4]. Li et al. and Amasheh et al. have reported down-regulation of claudin-1, claudin-3, claudin-5, claudin-7 and claudin-8 in UC [1], [12]. Another study by Oshima et al. showed a reduced expression of claudin-4 and claudin-7 in UC, whereas expression of claudin-2 was increased, as claudin-1 and claudin-3 expression levels were unchanged in controls and active UC [13]. Moreover, Mennigen et al. have recently demonstrated that expressions of TJ proteins such as claudin-1, claudin-3, claudin-4 and claudin-5 were decreased in acute colitis [14]. So far, only a few researches concert on the expression patterns of claudins in UC, and the results are still controversial, thus needs further investigation.

CXCR4 is specific receptor for the chemokine CXCL12, and also functions as an entry receptor for human immunodeficiency virus [15]. Early studies showed that the CXCL12/CXCR4 chemokine axis is involved in several inflammatory diseases such as rheumatoid arthritis, acute lung injury, and sepsis [16]–[19]. Recent studies demonstrated that CXCL12 and CXCR4 are constitutively expressed on intestinal epithelial cells, lamina propria T cells, and peripheral blood T cells of control patients, and the expression is increased in those of UC patients [20], [21]. Mikami et al. previously reported that blocking of CXCR4 significantly ameliorates mice experimental colitis, and the effect was partially dependent on the reduction of migration and cytokines production from mesenteric lymph node cells [22]. Whether CXCR4 antagonist has action on intestinal barrier and the possible mechanism, is largely undefined.

In the present study, we firstly assessed the effects of CXCR4 antagonist AMD3100 on cytokines, intestinal barrier, and colonic claudins expression in DSS-induced colitis in mice. To further elucidate the role of CXCR4 in intestinal barrier function, we also investigated the effects of CXCL12, AMD3100, and cytokines on claudin expression in HT-29/B6 colonic cells.

## Materials and Methods

### Materials

Dextran sulfate sodium (DSS, 5000 Daltons) was purchased from Wako Pure Chemical Industry (Osaka, Japan). CXCR4 antagonist AMD3100, fluorescein isothiocyanate conjugated dextran (FD4, 4000 Daltons), and fluorescent DNA-binding dye Hoechst-33342 were purchased from Sigma (St. Louis, MO, USA). Primary antibodies such as rabbit anti-claudin-1, claudin-2, claudin-3, claudin-5 and rabbit anti GAPDH were purchased from Abcam (OFW, UK). Rabbit anti-claudin-7 and claudin-8 were purchased from Zymed Laboratories (South San Francisco, CA, USA). Horseradish peroxidase-conjugated secondary antibody was purchased from Kangchen Biotech (Shanghai, China). Chemiluminecent HRP substrate was purchased from Millipore (Boston, MA, USA). Power vision two-step histostaining reagent was purchased from ImmunoVision Technologies (Norwell, MA, USA). HT-29 colonic cells were purchased from American Tissue Type Culture Collection (Rockville, MD, USA). CXCL12, tumor necrosis factor-α (TNF-α), interleukin-6 (IL-6), and interferon-γ (IFN-γ) were purchased from Chemicon International (Temecula, CA, USA). DMEM/F12 culture medium and fluorescein isothiocyanate (FITC) conjugated goat anti-rabbit IgG were purchased from Invitrogen (Carlsbad, USA). Trans-well bicameral chambers with 8 µm pores were purchased from Greiner Bio-One (Frickenhausen, Germany).

### Animals

Female BALB/c mice (9 weeks of age, weighing 20∼22 g) were obtained from the Animal Facility of the Jinling Hospital (Nanjing, China). Animals were housed under controlled temperature, humidity and day-night cycles, with free access to standard laboratory feed and water. Experiments were carried out in accordance with the Guidelines laid down by the NIH in the USA regarding the care and use of animals for experimental procedures (NIH publication No. 86-23, revised1985). The Animal Studies Ethics Committee of Jinling Hospital approved the experiments (approval ID, 2009065).

### Experimental colitis

For the induction of colitis, mice were given 5% DSS in their drinking water for 7 days. Control mice received regular drinking water throughout the experiment. Twenty-five micrograms of AMD3100 dissolved in 200 µl of phosphate-buffered saline (PBS) or 200 µl of PBS alone were administered intraperitoneally once daily during the study period. Eight mice were studied in each experimental group. On day 8, all mice were anesthetized with intraperitoneal administration of ketamine (50 mg/kg) and acepromazine (2 mg/kg), and the intestinal segments from the ileocecal valve to the anus (5∼6 cm in length) were collected for subsequent assays.

### Clinical Scores

Clinical scores (also mentioned as disease activity index, DAI) were determined by assessing the degree of body weight loss, stool consistency, and detection of fecal blood, as previously described [23], [24]. Body weight, rectal bleeding and stool consistency were monitored daily. For each parameter a score of 0 to 4 was attributed, giving rise to maximal score of 12. Weight loss score: 0 =  no weight loss; 1 = 1%–3% weight loss; 2 = 3%–6% weight loss; 3 = 6%–9% weight loss; 4≥9% weight loss. Stool consistency score: 0 =  normal; 2 =  loose stools; 4 =  watery diarrhea. Fecal blood score: 0 =  normal; 2 =  slight bleeding; 4 =  gross bleeding.

### Colonic cytokines and morphology examination

The distal segments of the colon (2 cm from the anal verge) were fixed in 10% neutral buffered formalin, and embedded in paraffin wax. The sections were cut at a thickness of 4 µm, deparaffinized with xylene, stained with hematoxylin and eosin (H&E), and examined by two experienced pathologists in a blinded fashion. The following morphological criteria were considered: score 0, no damage; score 1 (mild), focal epithelial necrosis; score 2 (moderate), diffuse necrosis of the villi; score 3 (severe), necrosis with neutrophil infiltrate in the submucosa; score 4 (highly severe), widespread necrosis with massive neutrophil infiltrate and hemorrhage [25].

The colonic levels of TNF-α, IL-6, and IFN-γ were evaluated using commercial colorimetric kits according to the manufacturer's instructions. The tissue homogenate enzyme-linked immunosorbent assay was determined with respect to the concentration of protein.

### Immunohistochemical staining of colonic claudins

Colonic sections (4 µm) were dewaxed in graded alcohols, and washed with tap water. Endogenous peroxidase activity was blocked with 3% (v/v) H_2_O_2_, and antigen was retrieved with microwave in 0.01 mol/L citrate buffer. The sections were then washed with 0.1 mol/L PBS. Rabbit anti-claudin-1, claudin-2, claudin-3, claudin-5, claudin-7 and claudin-8 were all applied at 1∶100 and incubated overnight at 4°C. Sections were washed in PBS, 20 min for four times. Power vision two-step histostaining reagent was used for detection. All sections were developed using diaminobenzidine and counterstained with hematoxylin.

### Western blot analysis

Western blot analysis was performed as previously described [1]. Total protein (20 µg) was separated from each sample by electrophoresis on a 4%∼20% SDS-polyacrylamide gel and electroblotted onto polyvinylidene difluoride membranes. Membranes were blocked in a blocking solution, incubated overnight with primary antibodies, and developed with a horseradish peroxidase-conjugated secondary antibody diluted at 1∶1000. Primary antibody was diluted as follows: claudin-1 at 1∶100, claudin-2 at 1∶200, claudin-3 at 1∶400, claudin-5 at 1∶200, claudin-7 at 1∶300, and claudin-8 at 1∶200. The immune complexes were then visualized on X-ray film using chemiluminecent HRP substrate. Additional immunoblots were performed using GAPDH antibody as the primary antibody to evaluate equal loading.

### Intestinal permeability measurement

Intestinal permeability was assessed by the mucosal-to-serosal clearance of FD4 in everted gut sacs, as described in previous studies [26], [27]. Intestinal segments from the ileocecal valve to the anus (5∼6 cm in length) were excised, and prepared in ice-cold modified Krebs-Henseleit bicarbonate buffer [26]. The intestinal segment was lavaged with 3 ml of PBS to remove fecal material, and then closed at one end with a 4–0 silk ligature. The gut sac was everted using a thin metal rod, then connected to a 1 ml syringe containing 0.4 ml of the KHBB solution, and secured with a 4–0 silk ligature 4 cm from the tip. The everted gut sac was gently distended with 0.4 ml of KHBB, suspended in a 100 ml beaker containing FD4 (20 µg/ml) in KHBB, maintained at 37°C in a water bath, and continuously bubbled with a gas mixture containing 95% oxygen and 5% CO_2_.

At the beginning of the incubation, a 1 ml sample was withdrawn from the beaker to determine the initial external (mucosal surface) FD4 concentration (FD4muc). After 30 min incubation, the gut sac was removed from the beaker, its diameter (D) and length (L) were measured, and the fluid on the serosal side was aspirated into the syringe to determine the FD4 concentration (FD4ser). The serosal and mucosal samples were centrifuged for 10 min at 1,000 g. One hundred microliters of the supernatant was diluted with PBS (900 µl), and fluorescence was measured (λex = 492 nm, slit width  = 1.5 nm; λem  = 515 nm, slit width  = 10 nm) in a spectrofluorometer (model F-7000, Hitachi, Japan). The mucosal-to-serosal clearance of FD4 was calculated using the following equations:

Mucosal surface area (A)  =  πLD

Mass of FD4 in the gut sac after 30 min incubation (M)  =  (FD4ser) ×0.4

Mucosal-to-serosal permeation rate of FD4 (PR, ng/min)  =  M/30 min

Mucosal-to-serosal clearance of FD4 (C, nl/min/cm^2^)  =  (PR/FD4muc)/A

### Cell culture

HT-29/B6 cells, which were selected from HT-29 cells differentiated by glucose-free culture, were used at passages 28–30 [28]. HT-29/B6 cells were cultured in Trans-well bicameral chambers (8 µm pores) with DMEM/F12 medium containing 10% fetal calf serum, 100 U/ml penicillin and 100 µg/ml streptomycin at 37°C in an atmosphere of 5% CO_2_ at a relative humidity of 90%.

### Immunofluorescent staining of claudins in HT-29/B6 cells

Seven days after seeding, the HT-29/B6 cells were treated with CXCL12 (50 ng/ml), AMD3100 (20 µmol/L), TNF-α (100 ng/ml), IL-6 (100 ng/ml), or IFN-γ (100 ng/ml) for 24 h with untreated cells as control. Cells were then fixed with 4% formaldehyde, permeablized with 0.1% Triton X-100, blocked with 1% BSA in PBS for 1 h at room temperature, and incubated overnight with rabbit polyclonal anti-claudin-3, claudin-5, claudin-7, and claudin-8 antibodies at 4°C. The immune complexes were developed with FITC-conjugated goat anti-rabbit IgG and visualized by fluorescent microscopy (model IX71, Olympus, Japan) with 488 nm filters. In all cases, cellular viability was >95% by trypan blue exclusion assay prior to use.

### Measurement of Apoptosis

Cytokine-induced apoptosis in HT-29/B6 cells was assessed using a fluorescent DNA-binding dye Hoechst-33342 [29]. Briefly, cells were cultured in Trans-well bicameral chambers and treated with cytokines (100 ng/ml TNF-α, 100 ng/ml IL-6, or 100 ng/ml IFN-γ) for 24 h with untreated cells as control. After washing twice with PBS, the treated and nontreated cells were fixed by adding 4% formaldehyde for 10 min, washed three times with pre-chilled PBS, and then stained with Hoechst 33342 (working concentration 5 µg/ml) in dark for 15 min. The cells were immediately washed with PBS and then examined using fluorescent microscopy. The characteristic apoptotic morphological changes were chromatin condensation and fragmentation.

To quantify apoptotic cells, HT-29/B6 cells were harvested after exposed to cytokines for 24 h, washed twice with cold PBS, resuspended in FITC-conjugated annexin V and propidium iodide (PI) for 10 min at room temperature in the dark., and analyzed by a FACScan flow cytometer (Becton Dickinson, NJ, USA).

### Monolayer Permeability Assays

Permeability studies were performed using confluent monolayers 14 days after seeding. The stock solution of permeability probe FD4 (25 mg/ml) was prepared by dissolving the compound in HEPES-buffered DMEM/F12 medium and passing it through a filter (0.45-µm pore size). For permeability studies, the medium on the apical side of the Trans-well chambers was replaced with 200 µl FD4 solution. The medium on the basolateral side of the Trans-well chambers was replaced with 500 µl of control medium or medium containing cytokines (100 ng/ml TNF-α, 100 ng/ml IL-6, or 100 ng/ml IFN-γ). After 24 h of incubation, 30 µl of medium was aspirated from apical or basolateral compartments for spectrofluorometric determination of FD4 concentration, as previously described [30], [31]. Measurements were made using a spectrofluorometer. Samples were diluted with 270 µl of diluted with PBS (900 µl), and fluorescence was measured (λex  = 492 nm, slit width  = 1.5 nm; λem  = 515 nm, slit width  = 10 nm). The permeability of the monolayers was expressed as a clearance (nl/cm^2^/h) as described previously [32].

### Migration Assays

Cells migration was investigated using a ‘scratch wound’ method [33]. HT-29/B6 cells were cultured to confluent cell monolayers and starved overnight in DMEM/F12 medium. Cells were carefully wounded using sterile 20-µl pipette tips. The wounded monolayers were washed twice with PBS to remove nonadherent cells and incubated at 37°C in complete media. The cells were then incubated in control medium or medium containing cytokines (100 ng/ml TNF-α, 100 ng/ml IL-6, or 100 ng/ml IFN-γ) for 24 h. The progress of migration was photographed immediately and again 24 h after wounding at the same location along the wound edges with an inverted microscope (model IX71, Olympus, Japan). The extent of healing was defined as the ratio of the difference between the original and the remaining wound areas versus the original wound area [34].

### Statistical analysis

Results are presented as mean and standard error of the mean (SEM). One-way repeated-measures ANOVA (followed by multiple pair-wise comparisons using the Student-Newman-Kleus method) were used for the analysis of differences between the experimental and control groups. All statistical analyses were carried out using the SPSS version 11.5 for Windows (Chicago, IL, USA), with statistical significance set at *P*<0.05.

## Results

### CXCR4 antagonist AMD3100 attenuated colonic damage and disease activity index in DSS-induced colitis

**Figure 1:**
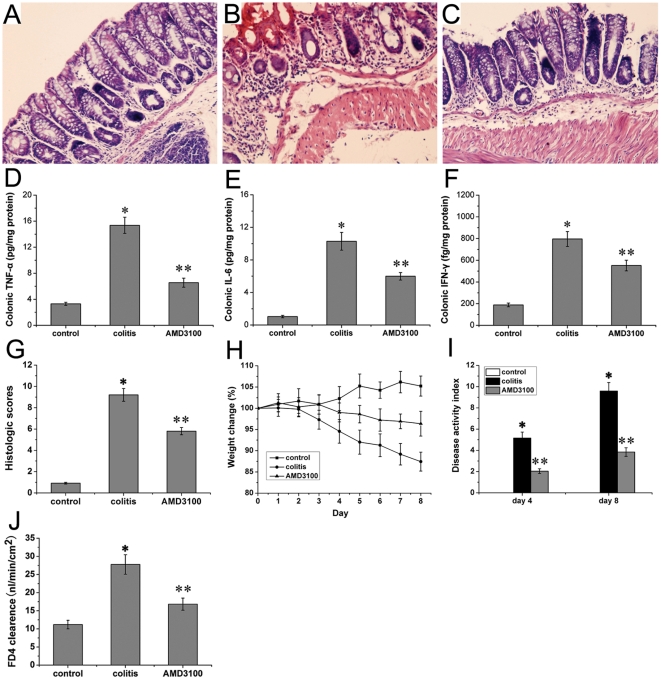
Effects of CXCR4 antagonist AMD3100 on colonic damage and disease activity index in mice. Colonic morphology from control groups (A), colitis group (B), and AMD3100 group (C) was examined in a blind-fashion. Colonic TNF-α (D), IL-6 (E), and IFN-γ (F) were evaluated by enzyme-linked immunosorbent assay. The histological score (G) was also determined. The weight loss (H), the fecal characteristics and fecal blood were monitored each day, and the disease activity index (I) was determined at day 4 and 8. The colonic epithelial barrier (J) was assessed using mucosal-to-serosal clearance of permeability probe FD4 in everted gut sacs. Eight mice were studied in each experimental group. Results are mean ± SEM. *P<0.05, colitis group *vs* control group; ** *P*<0.05, AMD3100 group *vs* colitis group. Original magnification, 200×.

After induction of colitis with DSS, the colonic mucosa showed congestion, erosion, and hemorrhagic ulcerations. Histological findings demonstrated marked epithelial destruction, inflammatory infiltration, and submucosal edema (Figure 1B). In AMD3100 treated mice, the epithelial destruction, inflammatory infiltration, and submucosal edema were markedly attenuated (Figure 1C). No histological alteration was observed in the intestinal segments from control mice (Figure 1A). Meanwhile, the levels of colonic TNF-α, IL-6, and IFN-γ in colitis mice were significantly higher than that in control mice, and treatment with AMD3100 markedly reduced the cytokines levels in colitis mice (Figure 1D-F). Accordingly, the histological score in mice with DSS-induced colitis was significantly higher than that in control mice, and treatment with AMD3100 markedly reduced the histological score in mice with colitis (Figure 1G).

Mice exposed to DSS for 7 days exhibited significant body weight loss (Fig. 1H) associated with diarrhea and fecal blood, giving rise to high DAI scores (Fig. 1I). Intraperitoneal injection of AMD3100 from days 1–7 of DSS exposure, prevented weight loss (Fig. 1H) and lowered DAI scores (Fig. 1I). These data indicate that CXCR4 antagonist AMD3100 could alleviate mucosal injury and clinical symptoms caused by DSS insult.

### AMD3100 enhanced intestinal barrier in DSS-induced colitis

The mucosal-to-serosal clearance of permeability probe FD4 in everted gut sacs was measured to assess the intestinal barrier. The mucosal-to-serosal passage of FD4 was low in control mice, and the calculated clearance was 11.18±1.17 nl/min/cm^2^. DSS-administered mice demonstrated a significant increase in gut permeability, with the calculated clearance reaching 27.77±2.70 nl/min/cm^2^. In AMD3100 treated mice, there was a marked reduction in gut permeability, and the calculated clearance was 16.81±1.67 nl/min/cm^2^ (Figure 1J).

### AMD3100 modulated the expression of colonic claudins in DSS-induced colitis

**Figure 2:**
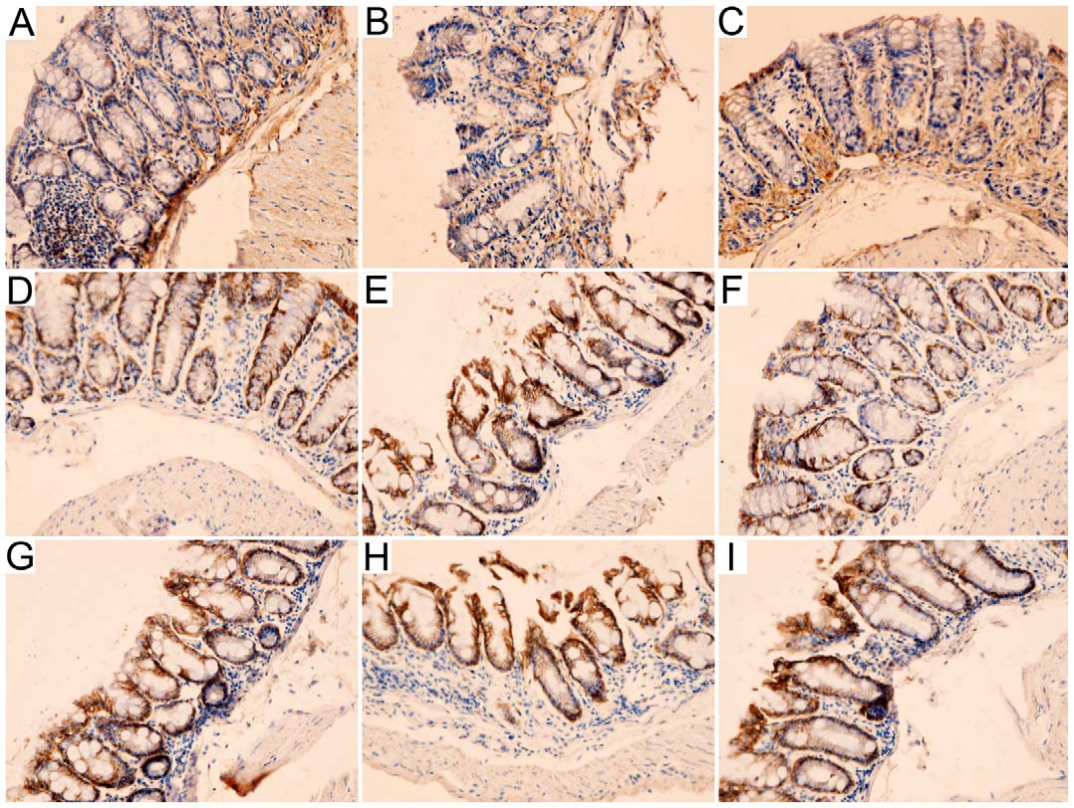
Representative photographs of colonic claudin-1, claudin-2 and claudin-3 immunostaining in mice. Moderate claudin-1 immunostaining was observed in colons from control group (A). The intensity was significantly decreased in colitis group (B), and increased when treated with AMD3100 (C). Intense claudin-2 and claudin-3 immunostaining was detected in control group (D, G). In colitis group, immunostaining of claudin-2 was increased in intensity (E), whereas claudin-3 immunostaining was decreased (H). Treatment with AMD3100 moderately reduced claudin-2 immunostaining (F) but enhanced claudin-3 immunostaining (I). Original magnification, 200×.

Immunolocalization of colonic claudins was investigated using immunohistochemical staining. Moderate claudin-1 immunostaining was observed in control group, which was predominantly distributed in colonic epithelium at the base of crypts, and smooth muscle cells at the submucous layer. Luminal colonic epithelium showed scattered immunostaining of claudin-1 (Figure 2A). The immunostaining of claudin-1 was decreased in intensity in colitis mice (Figure 2B), and enhanced when treated with CXCR4 antagonist AMD3100 (Figure 2C). Intense claudin-2 and claudin-3 immunostaining was detected in control group, which was predominantly distributed in colonic epithelium at the tip and lateral aspects of crypts (Figure 2D and G). In colitis group, immunostaining of claudin-2 was increased in intensity (Figure 2E), whereas the intensity of claudin-3 immunostaining was decreased (Figure 2H). Treatment with AMD3100 moderately reduced claudin-2 immunostaining (Figure 2F) but enhanced claudin-3 immunostaining (Figure 2I).

**Figure 3:**
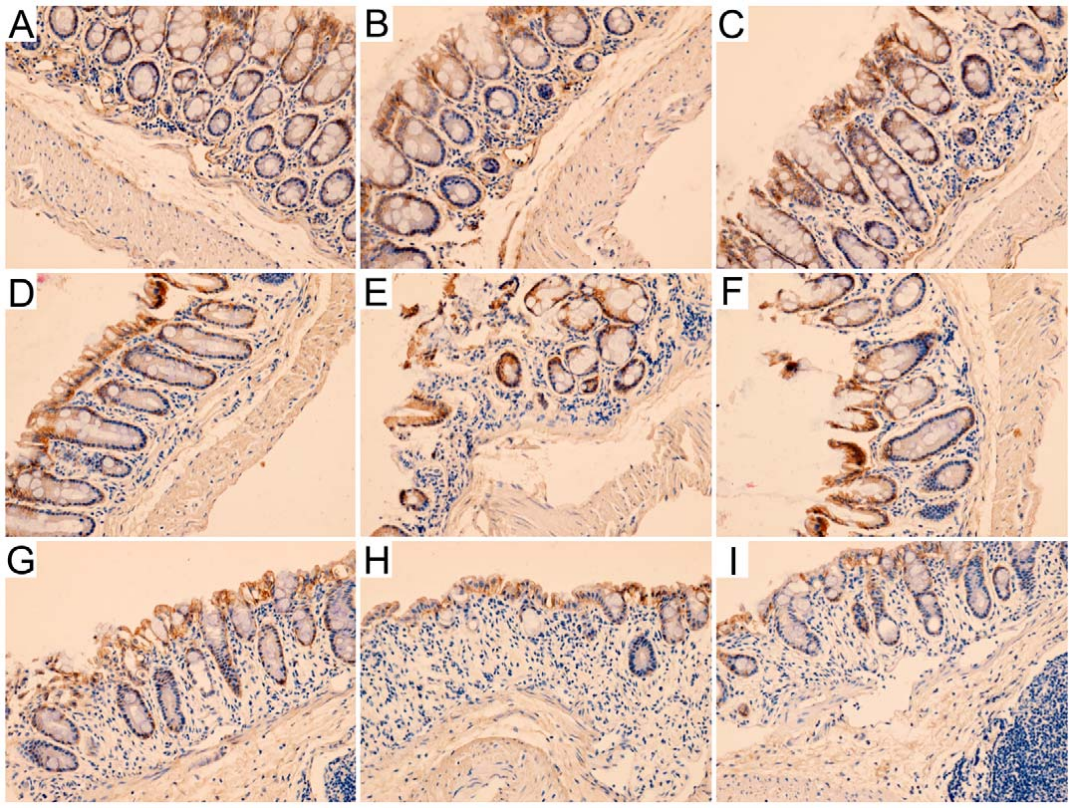
Representative photographs of colonic claudin-5, claudin-7 and claudin-8 immunostaining in mice. Intense claudin-5 immunostaining was observed in colons from control group (A). The intensity was significantly decreased in colitis group (B), and increased when treated with AMD3100 (C). Intense claudin-7 and moderate claudin-8 immunostaining were detected in sections from control group (D, G), and the intensity was markedly decreased in colitis group (E, H). Treatment with AMD3100 enhanced claudin-7 and claudin-8 immunostaining (F, I). Original magnification, 200×.

Intense claudin-5 immunostaining was observed in control mice, which was predominantly distributed in colonic epithelium at the tip and base of crypts, and colonic epithelium at lateral crypts showed scattered immunostaining of claudin-5 (Figure 3A). The immunostaining of claudin-5 was decreased in intensity in colitis mice (Figure 3B), and enhanced when treated with CXCR4 antagonist AMD3100 (Figure 3C). In control group, intense claudin-7 and moderate claudin-8 immunostaining were detected in colon, and predominantly distributed in colonic epithelium at the tip and lateral of crypts (Figure 3D and G). Intensity of claudin-7 and claudin-8 immunostaining was markedly decreased in colitis group (Figure 3E and H), and moderately elevated after treatment with AMD3100 (Figure 3F and I).

**Figure 4:**
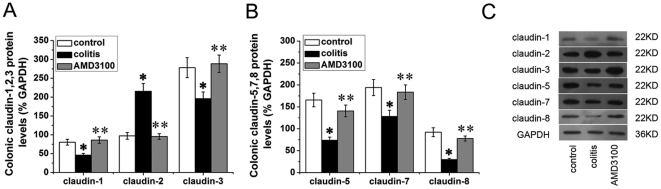
Effects of AMD3100 on colonic claudins expression in mice. Protein levels of colonic claudins were shown in (A). Representative immunoblots were shown in (B). Eight mice were studied in each experimental group. Results are mean ± SEM. *P<0.05, colitis group *vs* control group; ** *P*<0.05, AMD3100 group *vs* colitis group.

Protein levels of colonic claudins were accessed by western blotting. As shown in Figure 4, the expressions of colonic claudin-1, claudin-3, claudin-5, claudin-7 and claudin-8 in colitis mice were markedly decreased as compared with control mice. However, the expression of colonic claudin-2 was significantly increased in colitis mice. Treated with CXCR4 antagonist AMD3100 significantly promoted colonic claudin-1, claudin-3, claudin-5, claudin-7 and claudin-8 expressions, and also decreased colonic claudin-2 in colitis mice.

### AMD3100 had no influence on claudins expression in HT-29/B6 cells

**Figure 5:**
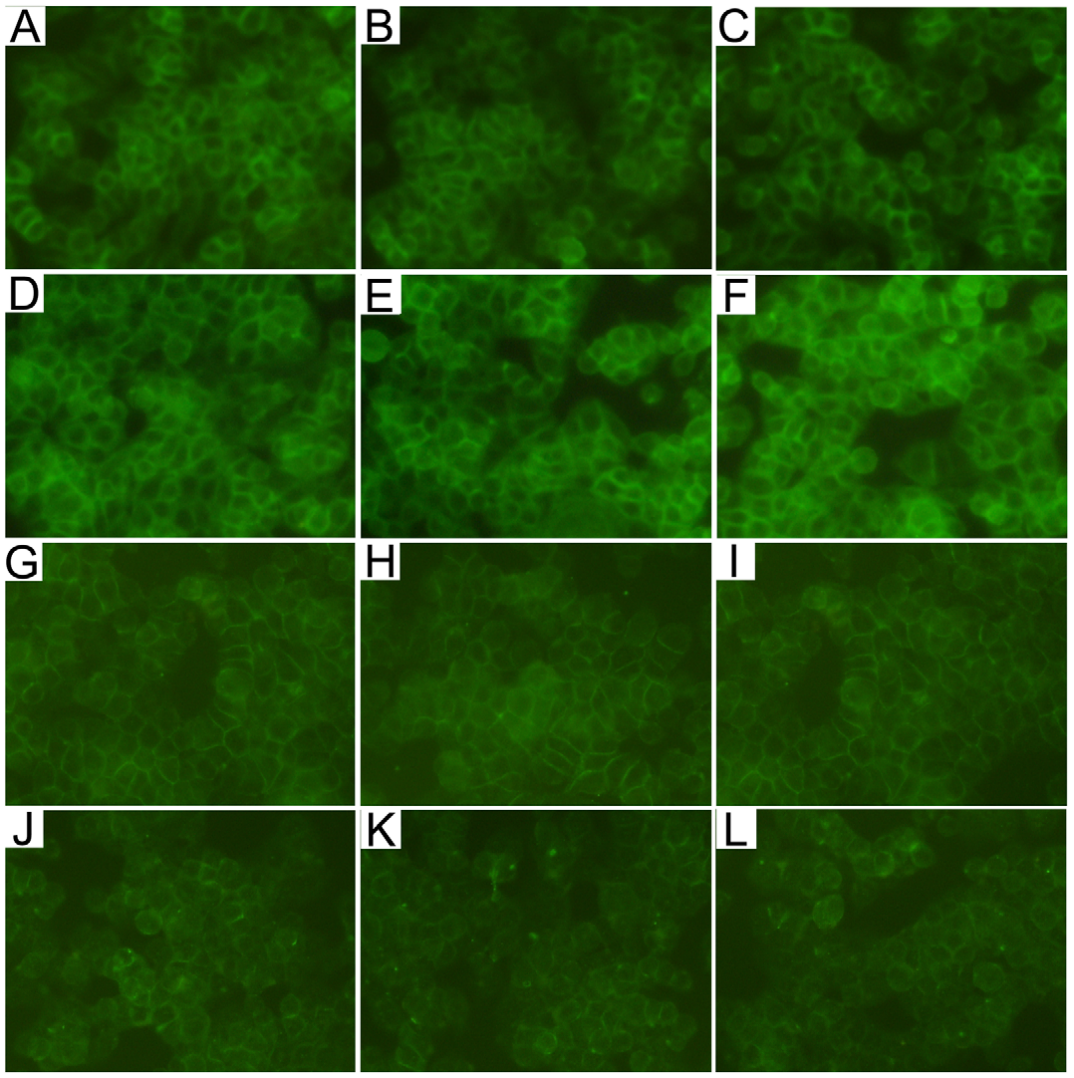
Representative photographs of claudin-3, claudin-5, claudin-7 and claudin-8 immunofluorescence in HT-29/B6 colonic cells. (A) claudin-3 in control group, (B) claudin-3 in CXCL12 group, (C) claudin-3 in AMD3100 group; (D) claudin-5 in control group, (E) claudin-5 in CXCL12 group, (F) claudin-5 in AMD3100 group; (G) claudin-7 in control group, (H) claudin-7 in CXCL12 group, (I) claudin-7 in AMD3100 group; (J) claudin-8 in control group, (K) claudin-8 in CXCL12 group, (L) claudin-8 in AMD3100 group. The immunofluorescence of claudin-3, claudin-5, claudin-7 and claudin-8 was predominantly distributed along the cellular membrane, and the immunofluorescent intensity remained unchanged after CXCL12 or AMD3100 treatment. Original magnification, 200×.

**Figure 6:**
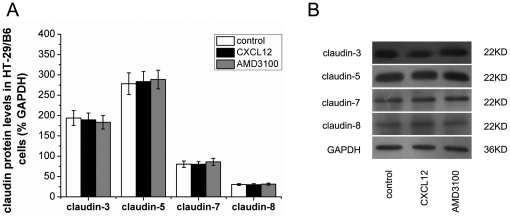
Effects of AMD3100 on claudins expression in HT-29/B6 colonic cells. Protein levels of claudins in HT-29/B6 cells were shown in (A). Representative immunoblots were shown in (B). Six wells were studied in each experimental group. Results are mean ± SEM.

Present study also investigated the expression of claudins in HT-29/B6 colonic cells treated with CXCL12 or CRXR4 antagonist AMD3100. Moderate claudin-3, claudin-7 and claudin-8, and intense claudin-5 immunofluorescence were detected in HT-29/B6 cells. The immunofluorescence was predominantly distributed along the cellular membrane (just the intercellular tight junction). Neither CXCL12 nor AMD3100 could influence the integrity and immunofluorescent intensity of claudins in HT-29/B6 cells (Figure 5). In accordance, western blotting analysis showed that protein levels of claudin-3, claudin-5, claudin-7 and claudin-8 in HT-29/B6 cells remained unchanged after CXCL12 or AMD3100 treatment (Figure 6).

### Cytokines altered claudin expression in HT-29/B6 cells

**Figure 7:**
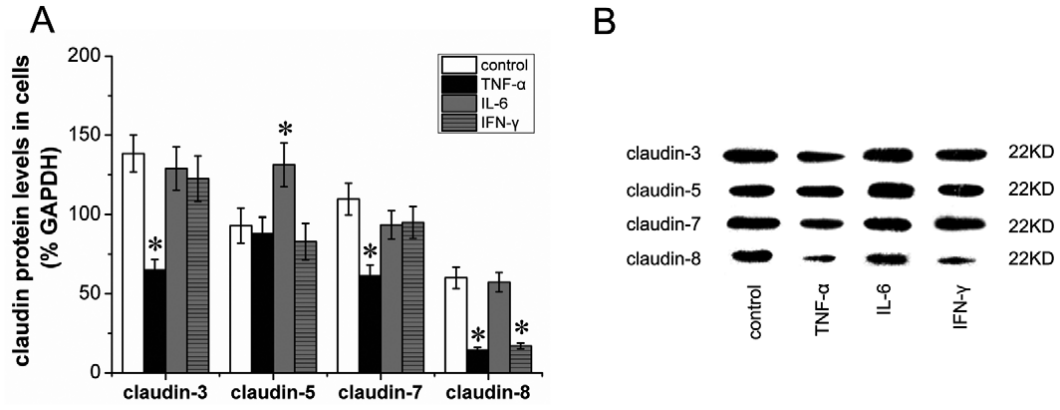
Effects of cytokines on claudins expression in HT-29/B6 colonic cells. Protein levels of claudins in HT-29/B6 cells were shown in (A). Representative immunoblots were shown in (B). Six wells were studied in each experimental group. Results are mean ± SEM. *P<0.05, *vs* control group.

Cytokines induced alteration of claudins was detected by western blotting. In HT-29/B6 cells, TNF-α treatment significantly decreased claudin-3, claudin-7, and claudin-8 levels as compared with control groups. Meanwhile, IFN-γ treatment markedly decreased claudin-8 levels. However, IL-6 treatment didn't alter claudin-3, claudin-7, and claudin-8 levels, but increased claudin-5 levels in HT-29/B6 cells (Figure 7).

### Cytokines inhibited wound healing in HT-29/B6 cells

**Figure 8:**
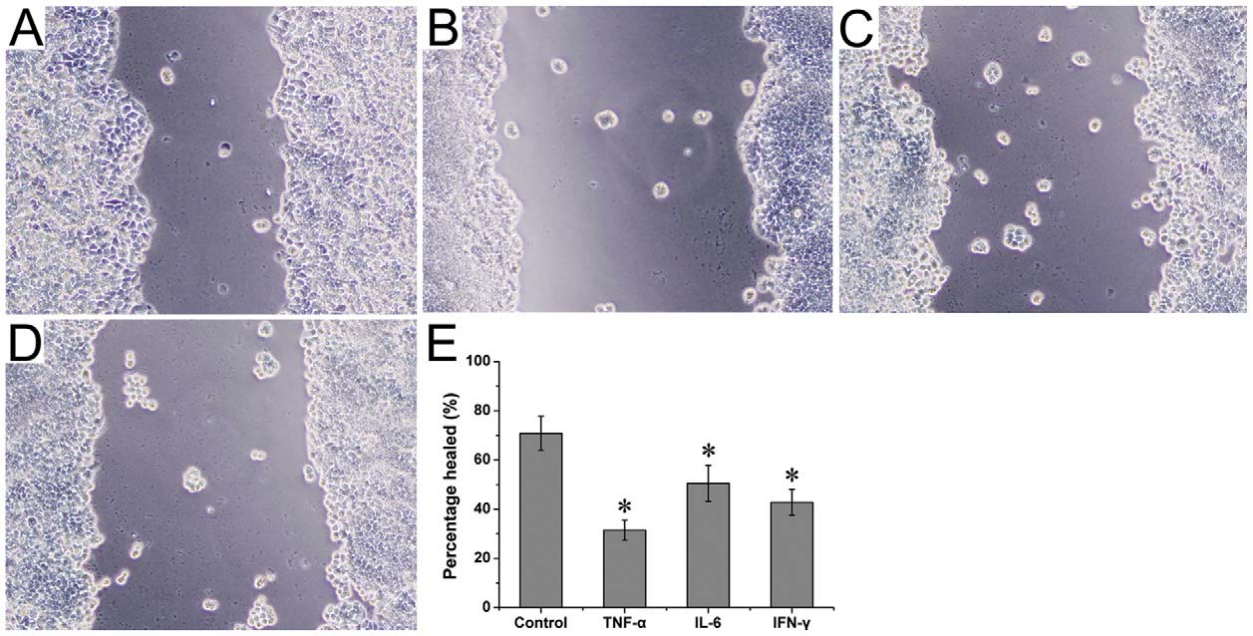
Effects of cytokines on wound-healing in HT-29/B6 colonic cells. (A) Control group, (B) TNF-α group, (C) IL-6 group, (D) IFN-γ group. The healing percentage was showed in (E). Six wells were studied in each experimental group. Results are mean ± SEM. *P<0.05, *vs* control group. Original magnification, 100×.

The wound healing was investigated using a ‘scratch wound’ method. As shown in Figure 8, the healed percentage in control group was 70.83±6.91. Treatment with TNF-α, IL-6, and IFN-γ significantly decreased the healed percentage (31.47±4.08, 50.52±7.35, 42.80±5.24, respectively). These results indicated that cytokines inhibited the cell migration, thus reduced wound healing in HT-29/B6 cells.

### Cytokines induced apoptosis and increased monolayer permeability in HT-29/B6 cells

**Figure 9:**
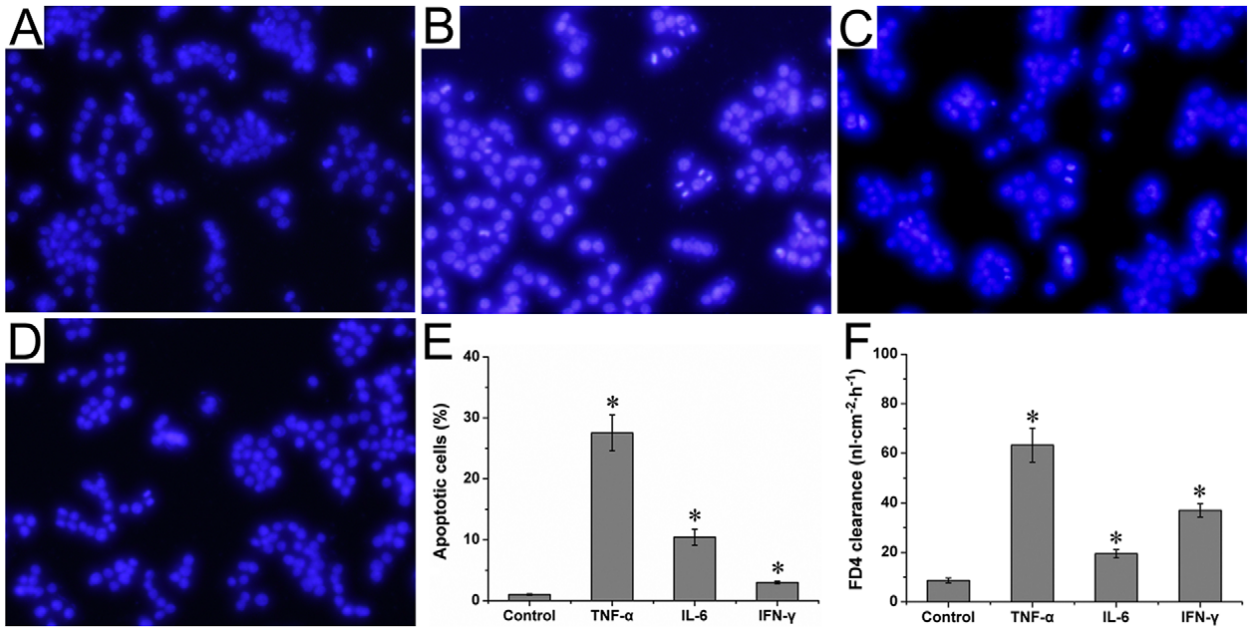
Effects of cytokines on apoptosis and monolayer permeability in HT-29/B6 colonic cells. (A) Control group, (B) TNF-α group, (C) IL-6 group, and (D) IFN-γ group showed respective apoptotic morphology. The apoptotic percentage was showed in (E). The monolayer permeability was expressed as a clearance of probe FD4, and was showed in (F). Six wells were studied in each experimental group. Results are mean ± SEM. *P<0.05, *vs* control group. Original magnification, 100×.

Apoptotic morphological changes were chromatin condensation and fragmentation. There were few apoptotic cells in control group (Figure 9A). Cytokines such as TNF-α, IL-6, and IFN-γ significantly increased apoptosis in HT-29/B6 cells (apoptotic percentage 27.52±2.94, 10.43±1.32, 2.98±0.23, respectively), as compared to control group (apoptotic percentage 1.01±0.11) (Figure 9B–E).

Monolayer permeability was expressed as a clearance of probe FD4. In confluent monolayers, TNF-α, IL-6, and IFN-γ significantly increased the FD4 clearance, as compared to control group (63.27±6.86 nl/cm^2^/h, 19.45±1.68 nl/cm^2^/h, 37.00±2.68 nl/cm^2^/h, respectively, vs 8.64±0.96 nl/cm^2^/h) (Figure 9F). These results indicated that cytokines increased the monolayer permeability in HT-29/B6 cells.

## Discussion

UC, known as inflammatory bowel disease, is characterized by activated mucosal immune system leading to impaired epithelial barrier function and tissue destruction [2], [35]. Intestinal epithelial barrier is maintained by intracellular junctional complexes, such as TJ, adherent junctions, and desmosomes [4]. Acting as intestinal barrier, TJ promotes the “fence” function that maintains the differential composition of the crypts by preventing the free diffusion of lipids and proteins between these compartments. By freeze fracture electron microscopy, previous investigations demonstrated reduction of TJ strands in UC, which is considered to be a possible cause of barrier dysfunction [4], [11]. Decrease of TJ complexes between epithelial cells disturbs the epithelial barrier, leads to increased intestinal permeability, which can facilitate the access of toxins and microbes to underlying tissues, and aggravate mucosal damage [11], [36], [37].

Additionally, the disrupted morphology of TJ is often the result of changes in TJ protein expression [4]. Claudins is the major integral membrane proteins forming the continuous TJ strands, interacting in a tissue-specific manner to form a charge-selective and size-selective barrier, and predominantly contributing to epithelial barrier function [6]–[10]. Disruption of claudins increases paracellular permeability, which may allow noxious contents to enter interstitium, and impairs alveolar epithelial barrier or blood-brain barrier, further aggravating local inflammation [38]–[41]. In UC, epithelial barrier function is also impaired. Li et al. have reported decreased expression of claudin-1, claudin-3, claudin-5 and claudin-8 in UC [1]. In accordance, Amasheh et al. recently demonstrated decreased expression of claudin-1, claudin-5 and claudin-7 in UC, whereas expression of claudin-2 was increased [12]. Other study by Oshima et al. showed a reduced expression of claudin-4 and claudin-7, and an increased expression of claudin-2 in UC, as claudin-1 and claudin-3 expression levels were unchanged in controls and active UC [13]. Moreover, Mennigen et al. have recently demonstrated that expressions of claudins such as claudin-1, claudin-3, claudin-4 and claudin-5 were decreased in acute colitis [14]. So far, researches concert on the expression of claudins in UC is only a few, and the results are still controversial.

In the present study, we identified the localization of claudin-1, claudin-2, claudin-3, claudon-5, claudin-7 and claudin-8 in colon tissue. By immunohistochemical staining, we found that claudin-1 was uniformly and continuously distributed in colonic epithelium at the tip and base of crypts, and in smooth muscle cells at the submucous layer in control mice. Moreover, claudin-2 and claudin-3 were predominantly distributed in colonic epithelium at the tip and lateral aspects of crypts, while claudin-5 was distributed in colonic epithelium along the crypts axis in control group. Furthermore, claudin-7 and claudin-8 were detected in colon of control mice, and predominantly distributed in colonic epithelium at the tip and lateral of crypts.

Present study also accessed the protein levels of claudins in colons by Western blotting. We found that DSS-induced colitis was associated with decreased expression of colonic claudin-1, claudin-3, claudon-5, claudin-7 and claudin-8, and also increased expression of colonic claudin-2. The change of colonic claudins was in parallel with aggravated mucosal damage and increased intestinal permeability. These results were consistent with studies from Li et al., Amasheh et al. and Mennigen et al.[1], [12], [14], and suggested possible role of claudins in intestinal barrier function. However, present study couldn't address whether the differential expression of claudins drives, or is a consequence, colitis.

Chemokines, which are expressed on various cells of the intestinal tissues, have been reported to regulate the recruitment of inflammatory cells [42], [43]. The chemokines CXCL12 is firstly characterized as a pre-B cell growth stimulating factor and its specific receptor is CXCR4, which also functions as an entry receptor for human immunodeficiency virus [15]. The CXCL12/CXCR4 chemokine axis is involved in several inflammatory diseases such as rheumatoid arthritis, acute lung injury, and sepsis [16]–[19]. Recent studies demonstrated that CXCR4 is constitutively expressed on intestinal epithelial cells and lamina propria T cells, and the expression is increased in those of UC patients [20], [21]. Block of CXCR4 significantly ameliorates murine experimental colitis [22], indicating a possible role of this CXCR4 in intestinal inflammatory response.

In present study, we demonstrated marked mucosal damage and inflammatory responses in DSS-induced colitis, and that could be ameliorated by CXCR4 antagonist AMD3100. Moreover, AMD3100 could prevent weight loss and lower DAI scores caused by DSS administration. These results are in agreement with a previous report that the CXCR4 antagonist, TF14016, could also ameliorate DSS-induced colitis [22]. Furthermore, our present study also demonstrated that AMD3100 could decrease intestinal permeability (indicated by reduced mucosal-to-serosal clearance of permeability probe FD4), thus enhance the intestinal barrier function. Present study identified the therapeutic effect of CXCR4 antagonist AMD3100 on experimental colitis.

Moreover, in the present study, we found that treated with CXCR4 antagonist AMD3100 significantly promoted colonic claudin-1, claudin-3, claudin-5, claudin-7 and claudin-8 expressions, and also decreased colonic claudin-2 in colitis mice. Although CXCL12 and CXCR4 constitutively expressing on intestinal epithelial cells [21], present study clearly demonstrated that neither CXCL12 nor CXCR4 antagonist AMD3100 could influence the integrity and protein levels of claudins in HT-29/B6 colonic epithelial cells. These results indicated that AMD3100 enhanced intestinal barrier function and modulated claudins expression through indirect pathways.

Previous studies had demonstrated that combination of TNF-α and IFN-γ could decrease claudin-3, claudin-5, and claudin-7 expression, with marked increase in paracellular permeability in rat colon [12], [44]. Moreover, Mazzon et al. reported that pharmacological and genetic TNF-α inhibition prevented the redistribution of claudin-5, and reduced the tight junction permeability *in vivo* [45], [46]. In the present study, we found that TNF-α could decrease the expression of claudin-3, claudin-7, and claudin-8 in HT-29/B6 colonic cells, while IFN-γ only decrease claudin-8 expression. Neither TNF-α nor IFN-γ could influence claudin-5 expression. These results were slightly different from the previous studies, probably attribute to the different targets (tissues *vs* cells).

Our present study also demonstrated that AMD3100 increased the expression of colonic claudin-1, claudin-3, claudon-5, claudin-7 and claudin-8, decreased of colonic claudin-2 expression in DSS-induced colitis. However, in HT-29/B6 colonic cells, TNF-α and IFN-γ decreased the expression of claudin-3, claudin-7 and claudin-8. Considering that AMD3100 could reduce TNF-α and IFN-γ production in vivo and in isolated lymphocytes [47], we speculated that CXCR4 antagonist AMD3100 acted on colonic claudins, at least partly, in a cytokine-dependent pathway.

Previous studies demonstrated that the maintenance of intestinal epithelial barrier was mainly dependent on the dynamic equilibrium of proliferation and in epithelial cells [48], [49]. Massive apoptosis of epithelial cells disturbed epithelial barrier, facilitated the infiltration of inflammatory cells, and aggravated mucosal damage [48]. In the present study, we found that TNF-α, IL-6, and IFN-γ increased apoptosis and monolayer permeability in HT-29/B6 cells. These cytokines also inhibited the wound-healing in HT-29/B6 cells. Increased apoptosis and delayed wound-healing of epithelial cells would augment monolayer permeability, and damage the epithelial barrier, as mentioned in previous study [48], [49].

In conclusion, the present study demonstrated that CXCR4 antagonist AMD3100 modulated the expression of colonic claudins, enhanced intestinal barrier function, also attenuated colonic inflammation in DSS-induced colitis. Considering the effects of cytokines on apoptosis, wound-healing, monolayer permeability, as well as claudin expression in vitro, we suggested that AMD3100 acted on colonic claudin expression and intestinal barrier function, at least partly, in a cytokine-dependent pathway.
